# Effectiveness, safety, and cost-effectiveness of thread-embedding acupuncture treatment for chronic rotator cuff disease: A randomized, patient-assessor-blinded, controlled, clinical trial

**DOI:** 10.1097/MD.0000000000046638

**Published:** 2026-01-02

**Authors:** Yeon-Cheol Park, Sangyeup Chae, Yeonhak Kim, Yoonsung Lee, Man S. Kim, Byung-Kwan Seo, Yong-Hyeon Baek

**Affiliations:** a Department of Acupuncture and Moxibustion, Kyung Hee University College of Korean Medicine, Kyung Hee University Hospital at Gangdong, Seoul, Republic of Korea; b Department of Acupuncture and Moxibustion, College of Korean Medicine, Kyung Hee University, Seoul, Republic of Korea; c Department of Clinical Korean Medicine, Graduate School, Kyung Hee University, Seoul, Republic of Korea; d East-West Bone and Joint Disease Research Institute, Kyung Hee University Hospital at Gangdong, Seoul, Republic of Korea; e Clinical Research Institute, Kyung Hee University Hospital at Gangdong, School of Medicine, Kyung Hee University, Seoul, Republic of Korea.

**Keywords:** chronic rotator cuff disease, cost-effectiveness, randomized controlled trial, shoulder pain, thread-embedding acupuncture (TEA)

## Abstract

**Background:**

Chronic rotator cuff disease (RCD) is a growing cause of persistent shoulder pain and disability. Although acupuncture is commonly used, sustained benefits remain uncertain. Thread-embedding acupuncture (TEA) provides prolonged stimulation, but evidence for chronic RCD is limited; thus, we evaluated its effectiveness, safety, and cost-effectiveness versus sham TEA.

**Methods:**

Patients with chronic rotator cuff disease were included. The treatments were divided into 2 groups once a week for 8 weeks. The control group received sham TEA, which was different from that administered to the experimental group. In other words, a randomized, patient-assessor-blinded, controlled, clinical trial was conducted. The effects of TEA were evaluated based on indicators related to pain reduction (100-mm visual analog scale), shoulder function improvement (shoulder pain and disability index, range of motion [ROM] of the shoulder), and quality of life (rotator cuff quality of life score and EuroQol 5-dimension 5 levels). Additional follow-up visits were conducted via telephone at 12 and 16 weeks of age.

**Results:**

The results showed pain relief and functional improvement within each group; however, no statistical significance was observed between each group. However, the ROM of the external rotation (ER) side was statistically significant in the experimental groups, as compared to the control group (*P* < .05). Adverse events that could be considered as safety issues have not been reported. In the cost-effectiveness evaluation, TEA treatment was found to be more economical, depending on the indices (i.e., some ROM indices and rotator cuff quality of life score) and the socially agreed willingness to pay based on those indices.

**Conclusion:**

The TEA treatment resulted in partial clinical improvements, including pain relief and shoulder function; however, these results were not statistically significant. Nevertheless, the TEA treatment was statistically significant in clinical improvement and cost-effectiveness for the range of external rotations.

## 1. Introduction

In modern society, the incidence of rotator cuff disease (RCD) is increasing, owing to aging and various sports activities. However, pain and economic loss in patients are also increasing due to the absence of effective treatments. RCD often results in chronic shoulder pain.^[1]^ The cause of RCD involves intrinsic factors of the rotator cuff itself and extrinsic factors triggered by the external environment or stimuli, which work in combination to trigger the symptoms. However, the causes and importance of these triggers remain unclear, hence leading to inconclusive diagnostic criteria. In general, conservative treatments, such as resting and strengthening of the rotator cuff muscles, are commonly used for RCD. In addition, medications^[2–4]^ or physical therapy^[5–7]^ as well as acupuncture^[8,9]^ and moxibustion^[10]^ are also commonly utilized.

Acupuncture has been widely used for diseases related to shoulder pain. According to a recent systematic review and meta-analysis, acupuncture showed potential benefits for shoulder pain including RCD^[11,12]^ and has been proven effective for pain relief and functional improvement in various pain diseases. The positive effects of acupuncture on chronic shoulder pain have also been reported in several studies.^[13–15]^ A systematic review suggested that acupuncture treatment for shoulder pain might be more advantageous than conventional drug therapies.^[16]^ However, some studies have reported that acupuncture can only temporarily reduce pain. Therefore, to maintain its effects for a relatively longer period of time, additional research on acupuncture may be needed.

Continuous acupuncture stimulation is an important treatment method for persistent pain; interest in thread-embedding acupuncture (TEA) has gradually increased in Korea. TEA treatment is related to sustained stimulation that produces a therapeutic effect on chronic pain and induces physiological inflammatory responses through the stimulation of soft tissues, improving the therapeutic effect and promoting regeneration responses.^[17]^ In the past, TEA was limited to cosmetic purposes, such as restoring skin elasticity, lifting facial muscles, reducing wrinkles,^[18,19]^ treating obesity,^[20]^ and treating the sequelae of facial palsy.^[21]^ Recently, many studies have reported its application in musculoskeletal diseases, such as herniated intervertebral disc^[22–24]^ degenerative knee osteoarthritis,^[25,26]^ low back pain,^[27,28]^ and myofascial pain syndrome.^[29]^ Localized pain is the most effective indication for TEA; however, various studies have reported that TEA can also be used when general acupuncture is less effective than expected, or when pain recurs frequently.^[30]^ Nevertheless, the scientific evidence for improving and reducing pain associated with TEA in specific diseases remains insufficient. Therefore, to verify the effects and safety of TEA and its use in clinical practice, a randomized controlled trial on diseases affecting many patients is required.

In the present study, chronic RCD was selected to evaluate the effects of TEA treatment, including pain reduction and tissue recovery, through continuous stimulation.^[31]^ Additionally, to confirm its effects, a sham thread-embedding acupuncture (STEA) group was used as the control group. A cost-effectiveness evaluation was conducted to assess the economic effects of TEA treatment. Finally, we attempted to confirm the effects, safety, and economic feasibility of TEA for the treatment of chronic RCD.

## 2. Materials and methods

### 2.1. Trial design

**Figure 1.**
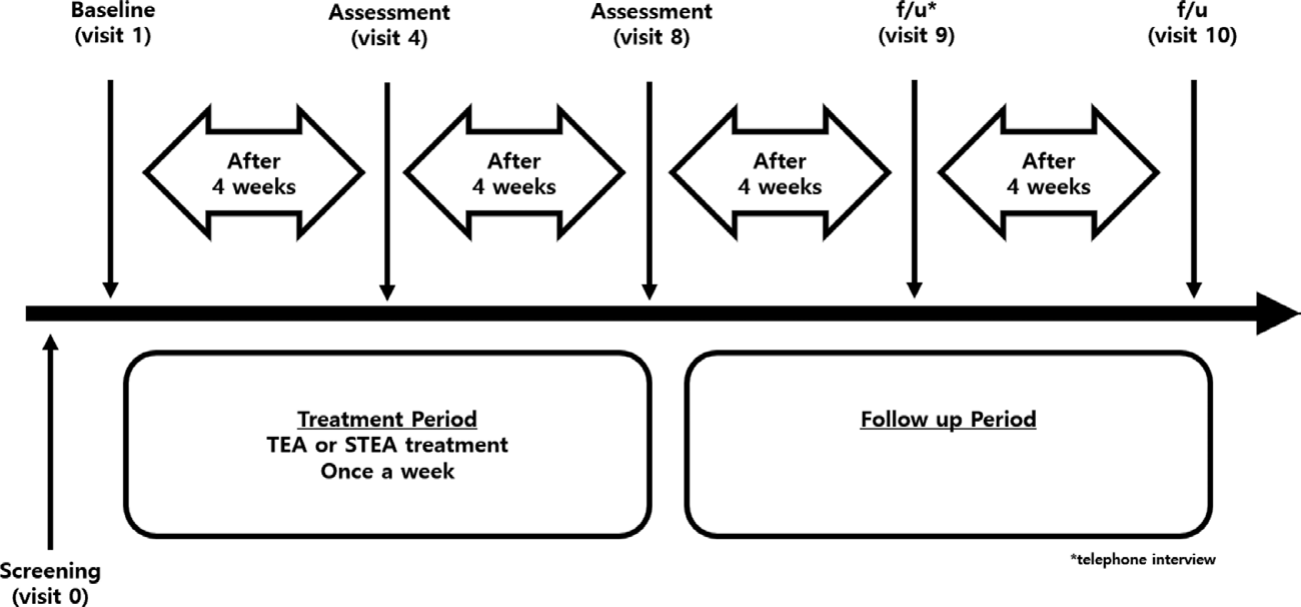
Trial design. f/u = follow-up, STEA = sham thread-embedding acupuncture, TEA = thread-embedding acupuncture.

This was a randomized, patient-assessor-blinded, sham-controlled trial with 2 arms. Sixty-four participants diagnosed with chronic RCD according to the criteria were randomly allocated to 2 groups in a 1:1 ratio; 1 group was allocated as the experimental group (TEA group) and the other as the control group (sham TEA group). The effects and safety of TEA were compared between the groups. The primary endpoint was 8 weeks after baseline. Participants were evaluated during a follow-up assessment at 12 weeks. The final assessment was performed 16 weeks after baseline, as illustrated in Figure 1.

### 2.2. Location and participants

The clinical trial was conducted from November 2017 to August 2018 at the Kyung Hee University Hospital, Gangdong. Recruitment notices were released on hospital websites and advertisement boards in the subway. All participants were interviewed via telephone and scheduled for a screening visit by the clinical research coordinator (CRC). The eligibility of the participants was evaluated by a Korean Medicine Doctor specializing in acupuncture and musculoskeletal disorders. Participants diagnosed with chronic RCD were referred to an independent investigator who performed the final eligibility assessment. After completing the screening test, participants were provided with detailed information. Prior to participant screening, a description of the study was distributed and fully explained to all applicants. The consent form was voluntarily signed by the participants; a copy was provided to each participant.

### 2.3. Eligibility criteria

The inclusion and exclusion criteria for chronic RCD were based on studies by Hermans^[31]^ and Bennell.^[32]^ Chronic RCD is a generic term used for diseases of the muscles or ligamentous fibrous membranes of the rotator cuff. It remains distinct from other diseases associated with shoulder pain in terms of causes, symptoms, prognosis, and treatment response.

### 2.4. Inclusion and exclusion criteria

Participants meeting the following inclusion criteria were recruited: 18 to 65 years; shoulder pain lasting > 3 months; severity of pain during activity (i.e., daily activity or light exercise) >3/10 on a 0 to 100-mm pain visual analog scale (VAS); a positive painful arc of abduction between 60° and 120° on pain provocation test; positive results in the empty can or external rotation resistance tests; ability to communicate sufficiently with the researcher; and agreement to not receive other treatments other than the prescribed 1 within the clinical trial period; and agreement to participate after providing written informed consent.

Participants with the following conditions were excluded: pain at rest >7/10 on a 0 to 100-mm pain VAS; >50% restriction of passive range of motion (ROM) in 2 or more shoulder planes; suspicion of rotator cuff tear based on the presence of a positive lag test on internal and external rotation; previous shoulder surgery history; radiological evidence of osteoarthritis, calcific tendinitis or previous fracture; systemic disease, including inflammatory joint disease or neoplastic disorder; radiating pain-related to cervical spine disease; intra-articular steroid injections within 3 months of participation in the clinical trial; having taken anti-inflammatory drugs or are currently taking them within 2 weeks of participation in the clinical trial; with a mental illness unable to fulfill the clinical trial compliance; and musculoskeletal disorders that may affect the effectiveness assessment, or any joint disease that renders the patient unable to participate in the clinical trial.

### 2.5. Dropout or early termination

Dropouts or early termination were defined as follows: if the participant administers drugs that are expected to affect safety and effectiveness, or are prohibited during the clinical trial period; if the participant requests suspension of the clinical trial, or withdraws consent to participate; if a serious adverse event (AE) occurs to the participant; if the participant violates the inclusion criteria or meets the exclusion criteria; if the participant receives less than 6 out of 8 treatment sessions; and if it is judged by the researcher in charge that the clinical trial should be stopped.

### 2.6. Randomization and allocation concealment

Randomization was implemented by an independent statistician using the website “www.randomization.com,” and enclosed in sequentially numbered, opaque envelopes. The envelopes were delivered to the research center and kept in a double-block cabinet. The interviewer (CRC) opened the envelope after the participants who met the eligibility criteria submitted their informed consent and allocated them to either the experimental or control group, according to the random numbers in the envelopes.

### 2.7. Blinding

Patient-assessor blinding was conducted because TEA could not be performed by the practitioner while blinded. Practitioners were prevented from having conversations other than those related to TEA or STEA. The participants were told that they would receive treatment in 1 of 2 different ways, namely, “classical thread-embedding therapy” and “nonclassical thread-embedding therapy.” All the participants received treatment under the same conditions as those used for acupuncture at a Korean Medical Clinic. Outcome measurements were performed by an independent assessor who did not perform the treatment or random assignment. The assessor simply asked about the content of the assessment and the case record, and then detailed the content without knowing what kind of treatment the participants received. The participants, assessors, statisticians, and related researchers were blinded to the allocation; blinding was maintained until the end of the clinical trial.

### 2.8. Study schedule of enrollment, allocation, and assessments

The study schedule is presented in Table 1. It comprises the screening, treatment, and follow-up phases. During the screening visit, the participants signed informed consent forms. The investigator conducted physical examination, medical history-taking, blood tests, shoulder radiography, initial VAS assessments, and concomitant treatment assessments to determine whether the participants were suitable for the clinical trial according to the eligibility criteria. Baseline measurements were obtained before the initial intervention. During the 8-week treatment period, participants received TEA or STEA treatment once a week from visits 1 to 8. During the follow-up period, the CRC contacted the participants 12 weeks after baseline (visit 9) via telephone interviews. Lastly, the participants visited the research institution for a final assessment 16 weeks after baseline (visit 10). The outcomes of shoulder pain, dysfunction, and quality of life were evaluated at 4 (visit 4), 8 (visit 8), and 16 weeks (visit 10). An inquiry into AEs and concomitant treatment was conducted at every visit. Treatment satisfaction was assessed at 8 (visit 8) and 16 weeks (visit 10).

**Table 1:** Clinical trial progress.

Trial schedule		Screening	Study period					Follow-up	
		V0	V1	V2–3	V4	V5–7	V8	V9†	V10
		−2 to 0W	1W	2–3W	4W*	5–7W	8W*	12W*	16W*
Getting participant consent		◎							
Demographic survey		◎							
Medical history and drug survey administered		◎							
Physical examination		◎							
Radiological examination		◎							
Subject eligibility assessment		◎							
Effectiveness assessment	100-mm Pain VAS	◎	◎		◎		◎		◎
	SPADI		◎		◎		◎		◎
	ROM		◎		◎		◎		◎
	RC-QoL		◎		◎		◎		◎
	EQ-5D		◎		◎		◎		◎
	Patient satisfaction assessment						◎		◎
	Economic evaluation						◎		◎
Safety assessment	Vital sign	◎	◎	◎	◎	◎	◎		◎
	Lab test	◎					◎		
	Concomitant drug survey		◎	◎	◎	◎	◎	◎	◎
	Adverse effect assessment		◎	◎	◎	◎	◎	◎	◎
Randomization			◎						
A confirmation of dropout			◎		◎		◎	◎	◎
Check visit date			◎	◎	◎	◎	◎	◎	◎
Experimental group/control group treatment			◎	◎	◎	◎	◎		

1) The Investigator checked the shoulder joint, performed physical examination (painful arc test, empty can test, external rotation resistance test, external rotation lag sign, and internal rotation lag sign), and checked for positivity.

2) Laboratory tests included erythrocyte sedimentation rate (ESR), blood urea nitrogen (BUN), creatinine (Cr), alanine aminotransferase (ALT), alanine transferase (AST), and C-reactive protein (CRP) levels.

3) Radiological examination was performed using shoulder X-ray 4P (axial, lateral, true AP, S-S outlet view); patients with osteoarthritis, calcified tendinitis, or fractures were excluded.

4) In the experimental group, TEA treatment was performed once a week; in the control group, STEA treatment was performed in a similar manner.

5) Previously taken anti-inflammatory analgesics were stopped during the treatment period.

ALT = alanine aminotransferase, AST = alanine transferase, BUN = blood urea nitrogen, Cr = creatinine, CRP = C-reactive protein, EQ-5D = EuroQol 5-dimension, ESR = erythrocyte sedimentation rate, RC-QoL = rotator cuff quality of life score, ROM = range of motion, SPADI = shoulder pain and disability index, VAS = visual analog scale.

* Allow ±3 d on the specified date.

† Perform a telephone interview on visit 9.

### 2.9. Intervention

In both groups, the intervention was administered once a week for 8 weeks by using a 29-gauge, 40-mm TEA or STEA needle (polydioxanone sutures, Hyundae Meditech, South Korea) on 6 predefined acupoints selected by an expert group according to the Standards for Reporting Interventions in Clinical Trials of Acupuncture (STRICTA) checklist (Table 2).^[33]^ All therapeutic procedures were performed by a Korean Medicine Doctor with over 15 years of clinical experience, who was trained in the acupuncture and moxibustion medicine residency program. No treatment other than the prescribed ones (TEA or STEA) was administered during the study period. Because TEA is administered via needle injection, disinfection was performed during the course of treatment. The treated area was thoroughly disinfected with sterile ethanol to prevent infection.

**Table 2:** Details of the thread-embedding acupuncture treatment based on the STRICTA 2010 checklist.

Item	Detail	
Acupuncture rationale	1a	Thread-embedding acupuncture
	1b	Based on textbook of acupuncture and moxibustion medicine & consensus of the KMD
	1c	All participants received standardized treatment
Details of needling	2a	TEA: 6, STEA: 6
	2b	[TEA] One transversal embedding from the LI16 to SI12 direction (Supraspinatus) One transversal embedding from the SI12 to SI13 direction (Supraspinatus) One transversal embedding from the SI11 to SI10 direction (Infraspinatus) One transversal embedding from the SI11 to SI12 direction (Infraspinatus) One transversal embedding from midpoint of LI15 and TE14 to center of deltoid (Deltoid) One perpendicular embedding on SI9 (Teres minor) [STEA] Performed in the same acupoints. Thread removed sham acupuncture used.
	2c	(1) Supraspinatus (2 points): Transverse embedding for 4 cm (2) Infraspinatus (2 points): Transverse embedding for 4 cm (3) Deltoid (1 point): Transverse embedding for 4 cm (4) Teres minor (1 point): Perpendicular embedding for 4 cm
	2d	De-qi
	2e	[TEA] Thread embedded [STEA] There is no lasting needle stimulation since the acupuncture needle is removed immediately after insertion.
	2f	[TEA] Thread embedded [STEA] There is no needle retention since the acupuncture needle is removed immediately after insertion.
	2g	29-gauge, 40-mm TEA or STEA needle (polydioxanone sutures, Hyundae Meditech, South Korea)
Treatment regimen	3a	8 sessions
	3b	Once a week for 8 weeks
Other components of treatment	4a	All other interventions for the shoulder pain including surgical procedures, acupuncture, cupping, moxibustion, herbal medicine, physical therapy, drug administered for the purpose of pain control (analgesics, muscle relaxants, antidepressants and anticonvulsant agent) are not allowed during the 8-week of the treatment phase (It does not apply to follow-up period). However, among all medicine taken 4 weeks prior to participation in the clinical trial, drugs that have no affect shoulder pain-related dysfunction are allowed under the investigator’s judgement.
	4b	The study was conducted at Korean Medicine Hospitals. Study participants were outpatients. All information except patient allocated group was provided to participants.
Practitioner background	5	Licensed KMDs with at least 15 years of acupuncture clinical experience treatment. The practitioners have completed the course of acupuncture specialist graduated from the University of Korean Medicine. Standardized operational procedures were written for practitioners to ensure identical treatment.
Control interventions	6a	Recent research on sham acupuncture has reported that simple insertion alone of the acupoint without any other needle stimulation could be effective. In this study, to confirm the embedding effect of thread, thread removed acupuncture needle was set as the control group so that the stimulation at the insertion of the needle was the same in both the experimental group and the control group. A control group was set up based on existing sham acupuncture studies and on textbook of acupuncture and moxibustion medicine, and the final decision was made through consensus of the KMDs.
	6b	All procedures of the control group including treatment period, number of treatments, acupoint and size of acupuncture were same as that of experimental group. However, thread removed TEA was used for control group instead of normal thread-embedding acupuncture.

KMD = Korean Medicine Doctor, STEA = sham thread-embedding acupuncture, STRICTA = standards for reporting interventions in clinical trials of acupuncture, TEA = thread-embedding acupuncture.

#### 2.9.1. TEA group (experimental group)

**Figure 2.**
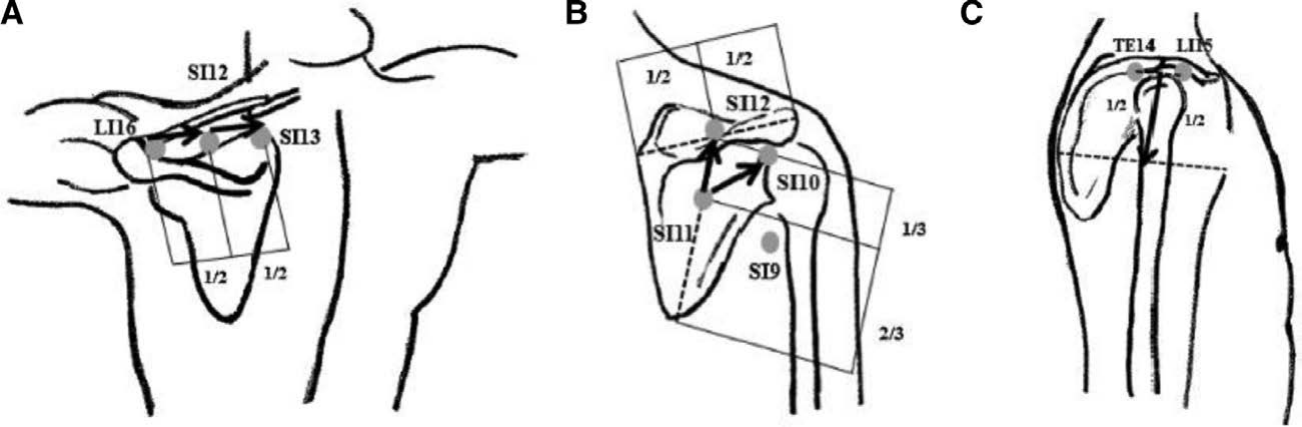
Acupoint and needle embedding direction. (A) Transverse thread-embedding direction for the supraspinatus muscle (LI16–SI12 and SI12–SI13). (B) Transverse thread-embedding direction for the infraspinatus muscle (SI11–SI10 and SI11–SI12) and perpendicular embedding point for the teres minor muscle (SI9). (C) Transverse thread-embedding direction for the deltoid muscle from the midpoint between LI15 and TE14 to the center of the deltoid.

Practitioners performed TEA at acupoints located on the 4 shoulder muscles closely related to chronic shoulder pain. The TEA procedure was expected to maximize not only the effect of the existing acupuncture treatment, but also the continuous stimulation effect on the major shoulder muscles. Therefore, transverse embedding was applied to the supraspinatus, infraspinatus, and deltoid muscles, whereas perpendicular embedding was applied to the teres minor muscles. In the supraspinatus muscle, 1 transverse insertion was applied from the LI16 (concave point between the acromial end of the clavicle and the spine of the scapula) to the SI12 (middle point of the supraspinatus fossa above the spine of the scapula), and another transverse insertion was made from SI12 to SI13 (the concave point of the inner end of the spine of the scapula; Fig. 2A). In the infraspinatus muscle, 1 transverse insertion was applied from SI11 (concave point between the upper one-third and lower two-thirds of the line connecting the midpoint of the scapular spine with the inferior angle of the scapula) to SI10 (concave point below the inner end of the scapular spine), and another transverse insertion from SI11 to SI12 (Fig. 2B). In the deltoid muscle, 1 transverse insertion was applied from the midpoint of LI15 (concave point between the outer edge of the acromion and the greater tubercle of the humerus) and TE14 (concave point between the acromial angle and the greater tubercle of the humerus) to the center of the deltoid muscle (Fig. 2C). In the teres minor muscle, 1 perpendicular insertion was applied to SI9 (approximately 3 cm from the back end of the axillary fold to the top; Fig. 2B). Details of the treatment group interventions are described in the STRICTA checklist (Table 2).^[33]^ When performing the TEA procedure, the suture of the insertion area was completely inserted, so that part of the suture was not exposed; however, if some sutures were exposed, the suture was removed and the procedure was repeated.

#### 2.9.2. STEA group (control group)

All procedures in the STEA group, including the treatment method, period, acupoints, number of treatments, and size of the TEA, were performed in the same manner as those in the TEA group, except that the thread was not inserted. Thread removal was performed aseptically and secretly to prevent infection and blinding.

### 2.10. Outcomes

Outcome measurements were evaluated by assessors who were blinded to group allocation. The primary outcome measure was shoulder pain intensity; the secondary outcome measures were pain intensity, shoulder disability, and quality of life.

#### 2.10.1. Primary outcome measurement (VAS)

Scores on the 100-mm pain VAS between baseline and treatment completion (8 weeks; primary endpoint) were used as the primary outcome measurements. “A painless state” was recorded as 0, and “the worst pain imaginable” was recorded as 100. It was marked in 3 conditions, “VAS at rest,” “VAS during motion,” and “VAS at night.” Participants were asked about the pain intensity they felt during the past 24 hours; they recorded the state themselves.

#### 2.10.2. Secondary outcome measurements

##### 2.10.2.1. The 100-mm pain VAS

The intensity of shoulder pain was assessed by determining the changes in the average 100-mm pain VAS score from baseline after 4 and 16 weeks, in the same way as the primary outcome measurement.

##### 2.10.2.2. Shoulder pain and disability index (SPADI)

Shoulder pain-related dysfunction was assessed using shoulder pain and disability index (SPADI) at baseline (visit 1), 4 weeks (visit 4), 8 weeks (visit 8), and 16 weeks (visit 10). The SPADI consisted of items divided into 2 subscales: 5 items for pain and 8 items for disability.^[34]^ Items were checked on a 10-point Likert scale (0 indicated “no pain” or “no difficulty”; 10 indicated “worst imaginable pain” or “so difficult it required help”). The SPADI score was calculated out of 100, with a higher score indicating greater pain/disability.

##### 2.10.2.3. Range of motion (ROM, shoulder)

Shoulder ROM was measured using a goniometer at baseline (visit 1), 4 weeks (visit 4), 8 weeks (visit 8), and 16 weeks (visit 10). The participants were examined for 5 motions, which included forward flexion (range, 0–180°), internal and external rotation at 90° shoulder abduction (range, 0–90°), external rotation at the side (range, 0–90°), and internal rotation posterior (behind the back; range, 0–90°).^[35]^

##### 2.10.2.4. Rotator cuff quality of life score (RC-QoL)

The RC-QOL (rotator cuff quality of life score) is a disease-specific, health-related, patient-reported outcome measurement that was developed for use in patients with the “full spectrum of RCD”; it was evaluated at baseline (visit 1) as well as after 4 (visit 4), 8 (visit 8), and 16 weeks (visit 10). The RC-QOL consists of 34 questions and 5 subscales: symptoms and physical complaints, 16 items; work-related concerns, 4 items; recreational activities, sports participation, or competition concerns, 4 items; lifestyle concerns, 5 items; and social and emotional concerns, 5 items.^[36]^ Each item was answered on a 100-mm VAS; a score of 0 implied “the most excruciating pain or discomfort”; a score of 100 indicated “no pain or discomfort.” The lowest score was 0%, indicating the worst quality of life; the best quality of life or asymptomatic score was 100%.^[37]^

##### 2.10.2.5. EuroQol 5-dimension 5 levels (EQ-5D-5L)

The general health status of the participants was assessed using the EQ-5D-5L^[38,39]^ at baseline (visit 1) as well as after 4 (visit 4), 8 (visit 8), and 16 weeks (visit 10). The EQ-5D-5L consists of 5 questions on morbidity, personal care, daily activities, pain/discomfort, and anxiety/depression. Each question was rated from 1 to 5 (1 = no problems; 2 = slight problems; 3 = moderate problems; 4 = severe problems; and 5 = extreme problems). The EQ-VAS is used to assess a patient’s health status. It is a 20-cm scale numbered from 0 to 100; a score of 0 indicates “the worst health condition imaginable”; a score of 100 indicates “the best health condition imaginable.”^[40]^

#### 2.10.3. Safety outcomes

The investigator checked for the AEs reported by the participants to monitor the safety of the intervention. An AE is an undesirable and unintended sign, symptom, or disease that occurs in a participant receiving an intervention used in a clinical trial. The investigator trained the participants to voluntarily report AEs and checked the AE at every regular or additional visit. The vital signs of the participants, including blood pressure, pulse, and temperature, were assessed at every visit. Changes in laboratory test values at baseline and after 8 weeks were also assessed. The investigator assessed the severity of each AE; the major AEs during the study were reported based on WHO guidelines, with the severity rated as mild (no restriction on temporary or routine activity), moderate (some restricted activities), or severe (severely restricted activities or when medical treatment was absolutely required). The causal relationship between the AEs and the intervention used in the clinical trial was categorized into one of the following 6 criteria: definitely related, probably related, possibly related, probably unrelated, definitely unrelated, or unknown. The investigator recorded all information related to the AEs in a case report form (CRF), including the type, dates of occurrence and disappearance, severity and results, and relevance to the drug and treatment.

#### 2.10.4. Economic evaluation

Cost-effectiveness was estimated by comparing TEA treatment (experimental) with simple acupuncture treatment (control). The effect indices were ROM, RC-QoL, and EQ-5D-5L; the outcome index was the incremental cost-effectiveness ratio (ICER). This study analyzed cost-effectiveness from a social perspective. In addition to the treatment costs, informal medical (over-the-counter medicine, health functional foods, and medical devices purchased for the purpose of managing personal health), transportation, nursing, and patient time costs were also estimated. Formal medical costs were obtained using “ingredients methods” to calculate the total cost by obtaining information on medical resource usage and unit costs; informal medical and nonmedical costs were obtained through survey responses and calculated using average wages information. The analysis period was based on the treatment period (8 weeks). The main effect indices and cost-related data obtained through clinical trials were separately analyzed using intention-to-treat (ITT) and per-protocol (PP) analysis methods. Cost-effectiveness was calculated as the ICER, which represented the economic value as compared with the additional effect of additional costs. If the experimental group had better effectiveness and lower costs than the control group, it was expressed as “Dominant”; if the experimental group had lower effectiveness but higher costs than the control group, it was expressed as “Dominated.”

### 2.11. Prohibited and permitted concomitant treatment

All other interventions for shoulder pain, including surgical procedures, acupuncture, moxibustion, herbal medicine, cupping, physical therapy, and drugs administered for pain control (analgesics, muscle relaxants, antidepressants, and anticonvulsant agents), were not permitted during the 8-week treatment period. However, among the drugs taken 4 weeks before participation in the clinical trial, those that did not affect shoulder pain-related dysfunction were allowed under the investigator’s judgment. Information on concomitant medications (i.e., product name, purpose of administration, dose, and duration of administration) was recorded on a CRF at each visit.

### 2.12. Data monitoring

Data collection was performed in accordance with the standard operating protocol of the KHUHGD Institutional Review Board (IRB); the quality of the study was managed by the Clinical Research Association (CRA) of an independent contract research organization (CRO). Monitoring included compliance assessment of the recruitment and intervention procedures with the protocol, as well as consistency evaluation between the records in the CRF and the original document. The monitoring process also managed and reported AEs that may have occurred during the clinical trials.

### 2.13. Sample size calculation

The sample size was calculated based on a previous study using the same primary outcome measurement (100-mm pain VAS) among randomized controlled clinical trials of acupuncture for RCD.^[41]^ According to the study, the standard deviation (SD) was 26.246. The mean difference before and after treatment was set at 20 mm, based on a study of minimal clinically important differences (MCID) in pain VAS scores for RCD.^[42]^ With a 5% significance level, 80% power, and 1:1 ratio, the sample size was calculated to be 28, using the formula below (assuming σ = 26.246 and *d* = 20):


$$n=\left(1+\lambda \right)\sigma^{2}{\left({\;Z\;}_{\alpha /2}+{\;Z\;}_{\beta }\right)}^{2}/\lambda d^{2}$$

Considering a 10% dropout rate, a total of 64 participants were required (32 participants per group).

### 2.14. Ethical approval

All participants voluntarily participated and could withdraw from the study at any time if desired. All data obtained during the clinical trial were recorded in a CRF; confidentiality was maintained. Clinical trials were conducted in compliance with the Korean good Clinical Practice (KGCP) and Declaration of Helsinki. The trial was approved by the IRB of KHUHGD (IRB No. KHNMCOH 2017-06-006) and registered with the Clinical Research Information Service (CRIS) of South Korea, which is registered as a Registry on the WHO International Clinical Trials Registry Platform (identifier: KCT0002563). The protocol for this study has been published in a research paper.^[43]^

### 2.15. Statistical analysis

An independent statistician blinded to the group allocation performed statistical analyses using the Statistical Package for the Social Sciences (SPSS) for Windows version 18.0; significance was set at *P* < .05. In the effectiveness analysis, both the ITT analysis and PP sets were used as follows: ITT analysis was the main analysis method, whereas the PP group was analyzed using the assisted analysis method. The primary outcome measure of this study was the mean change in the average 100-mm pain VAS scores acquired at baseline and 8 weeks. To validate significant changes between groups, changes in the VAS scores were expressed as the mean ± SD; independent *t*-tests were performed for comparisons between the groups. Trends over time and time-by-treatment interactions were analyzed using repeated-measures analysis of variance (ANOVA). In the secondary outcome analysis, continuous variables were analyzed in the same manner as in the primary outcome analysis. Binomial variables were presented as descriptive statistics (frequency and percentage); the chi-squared test or Fisher’s exact test was performed for comparisons between the groups.

## 3. Results

### 3.1. Participant analysis

**Figure 3.**
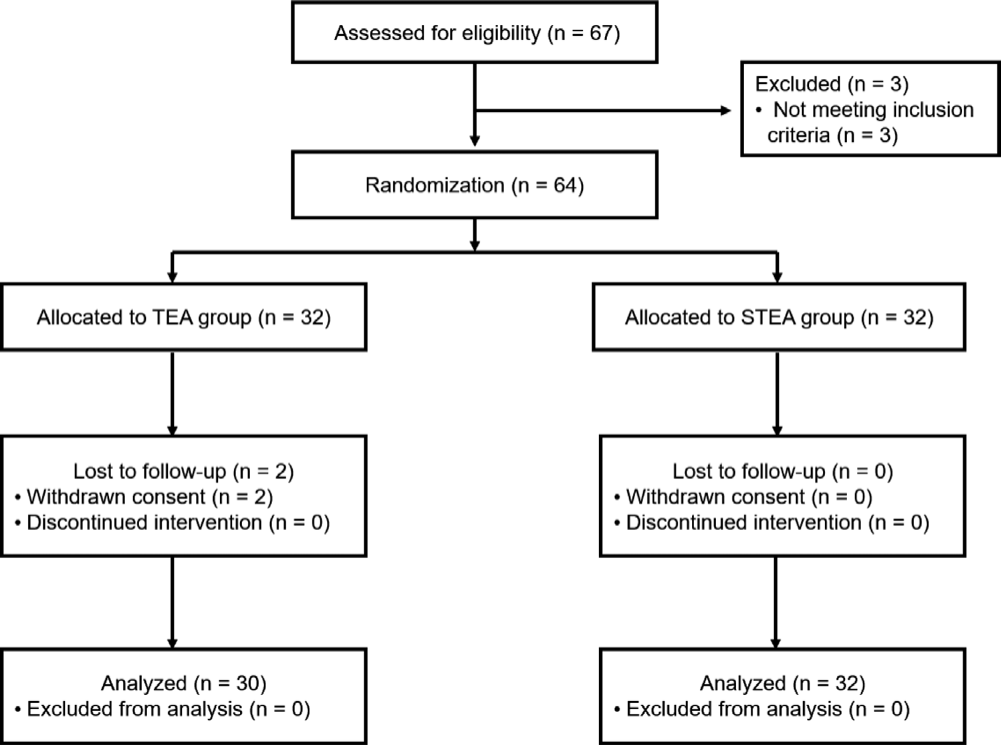
Flow diagram of the study. TEA = thread-embedding acupuncture, STEA = sham thread-embedding acupuncture.

From the recruitment discoveries for the clinical trial, 67 people were screened, of whom 64 were registered in the clinical trial. During the trial, 2 patients dropped out and 62 patients completed the clinical trial. Therefore, 64 people were included in the ITT analysis group; 62 people, excluding the 2 people who dropped out, were included in the PP analysis group. Two participants withdrew their consent; neither dropout was directly related to the trial procedure or assessment of validity and safety (Fig. 3).

For participants in the clinical trial, we checked whether there was a difference between the groups in terms of basic characteristics before the intervention. No statistically significant differences were observed in sex and age between the ITT and PP analysis groups; therefore, homogeneity between the groups was recognized. Randomization was judged to be statistically appropriate (Table 3).

**Table 3:** Participants’ baseline characteristics.

Variable	ITT population			PP population		
	Control (n = 32)	Experimental (n = 32)	*P* value	Control (n = 32)	Experimental (n = 30)	*P* value
Sex (male)	18 (52.3%)	16 (50.0%)	.616*	18 (52.3%)	16 (53.3%)	.818*
Age (yr)	51.38 ± 11.15	51.13 ± 9.96	.979†	51.38 ± 11.15	52.07 ± 10.27	.972†

ITT = intention-to-treat, PP = per-protocol.

* *P* values were derived from Chi-square test.

† *P* values were derived from Mann–Whitney’s *U* test (Shapiro–Wilk’s test was used for test of normality assumption).

### 3.2. Primary outcome measurement

#### 3.2.1. ITT analysis group

**Figure 4.**
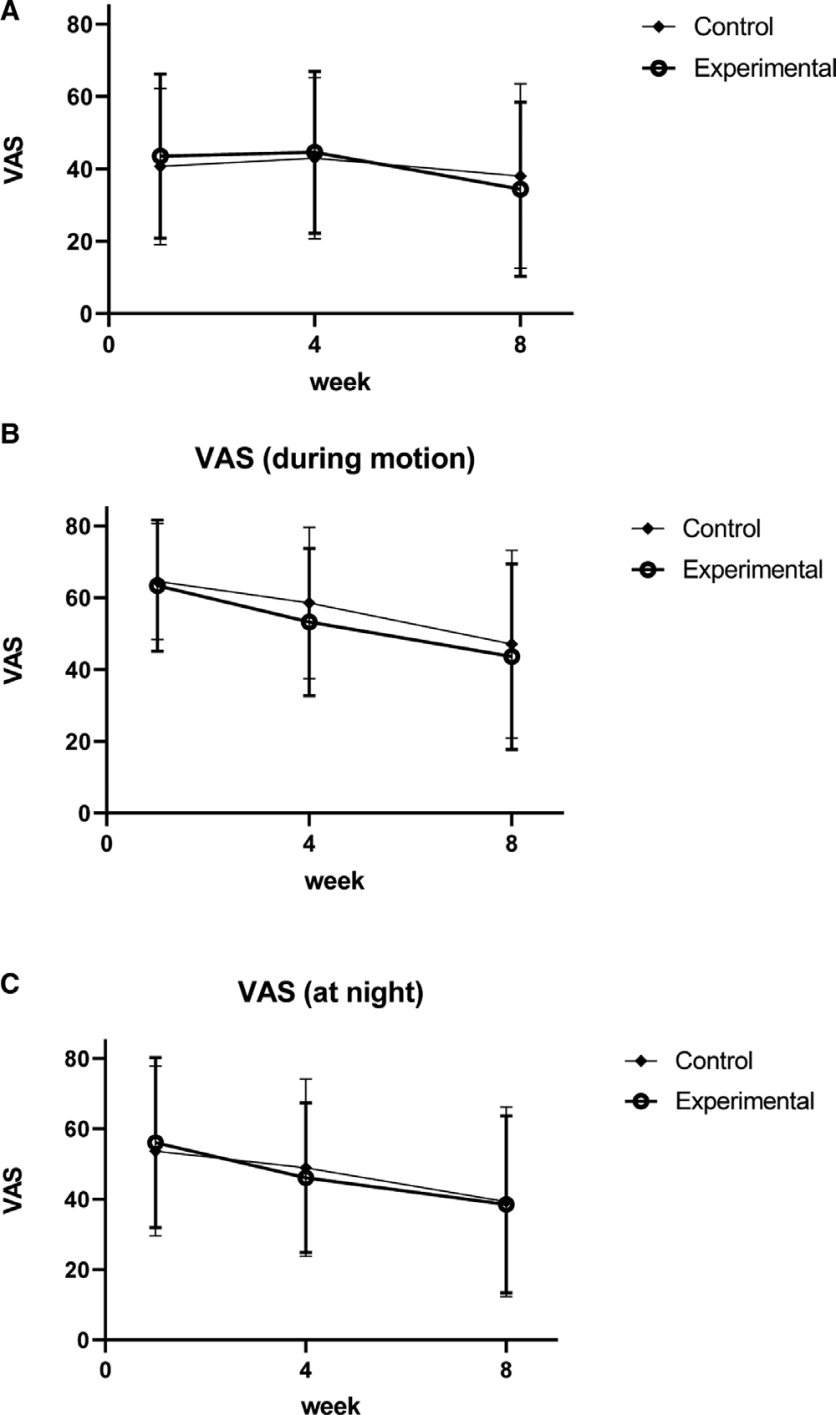
(A–C) Changes in visual analog scale (VAS; primary outcome measurement: intention-to-treat). VAS = visual analog scale.

The VAS pain scores significantly decreased in each group (*P* < .05); however, there was no statistically significant difference between the groups. However, when comparing the difference in variance of “VAS at rest” of the experimental (−9.16 ± 25.93) and control groups (−2.66 ± 26.47), “VAS during motion” of the experimental (−19.78 ± 22.60) and control groups (−17.47 ± 25.00), and “VAS at night” of the experimental (−17.59 ± 21.89) and control groups (−14.44 ± 23.53), the effect of pain relief in the experimental group was more noticeable than in the control group (Fig. 4A–C).

#### 3.2.2. PP analysis group

**Figure 5.**
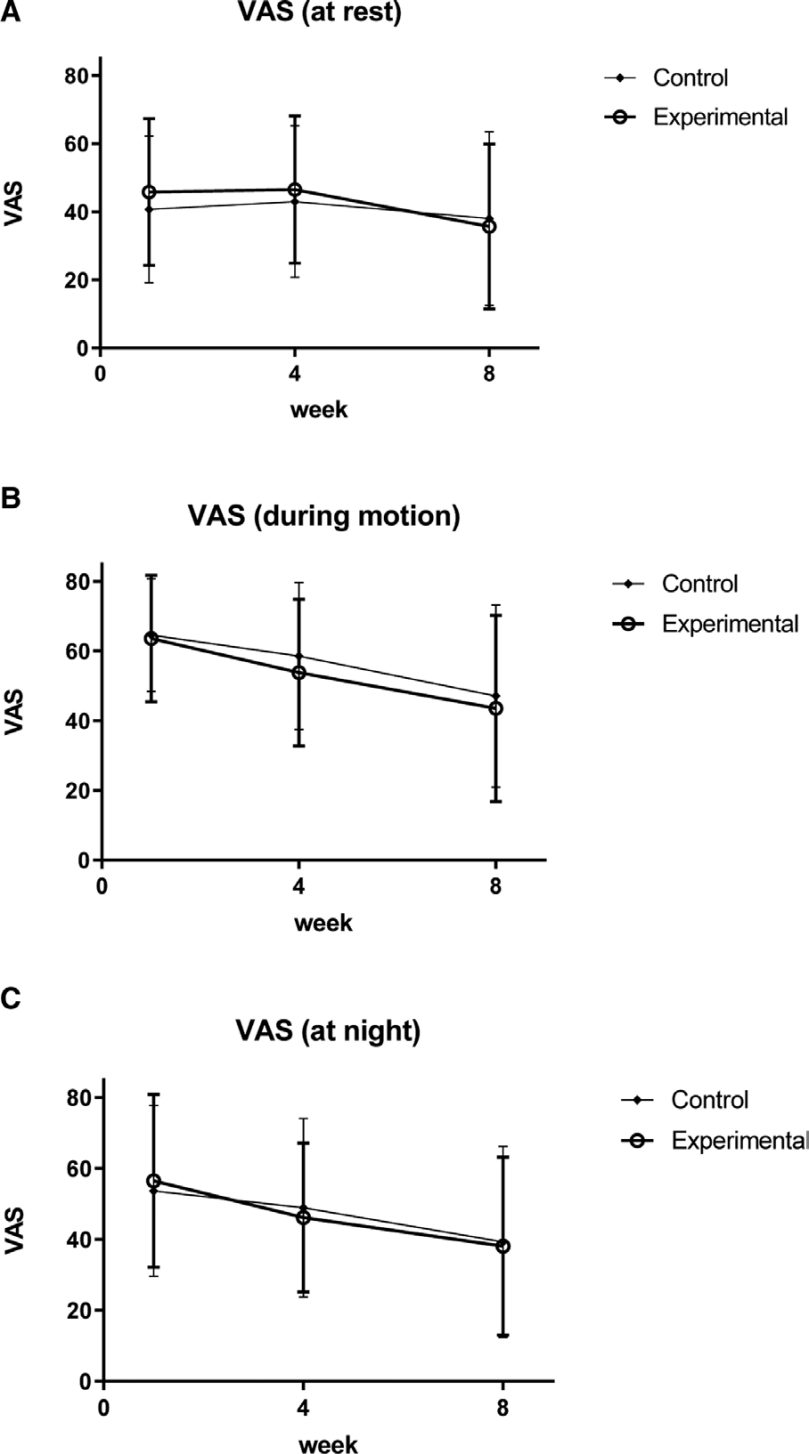
(A–C) Changes in visual analog scale (VAS; primary outcome measurement: per-protocol). VAS = visual analog scale.

The VAS pain scores significantly decreased in each group (*P* < .05); however, there was no statistically significant difference between the groups. However, when comparing the difference in variance of “VAS at rest” of the experimental (−10.10 ± 25.93) and control groups (−2.66 ± 26.47), “VAS during motion” of the experimental (−20.10 ± 22.99) and control groups (−17.47 ± 25.00), and “VAS at night” of the experimental (−18.43 ± 22.33) and control groups (−14.44 ± 23.53), the effect of pain relief in the experimental group was more noticeable than in the control group (Fig. 5A–C).

### 3.3. Secondary outcomes measurement

#### 3.3.1. ITT analysis group

**Figure 6.**
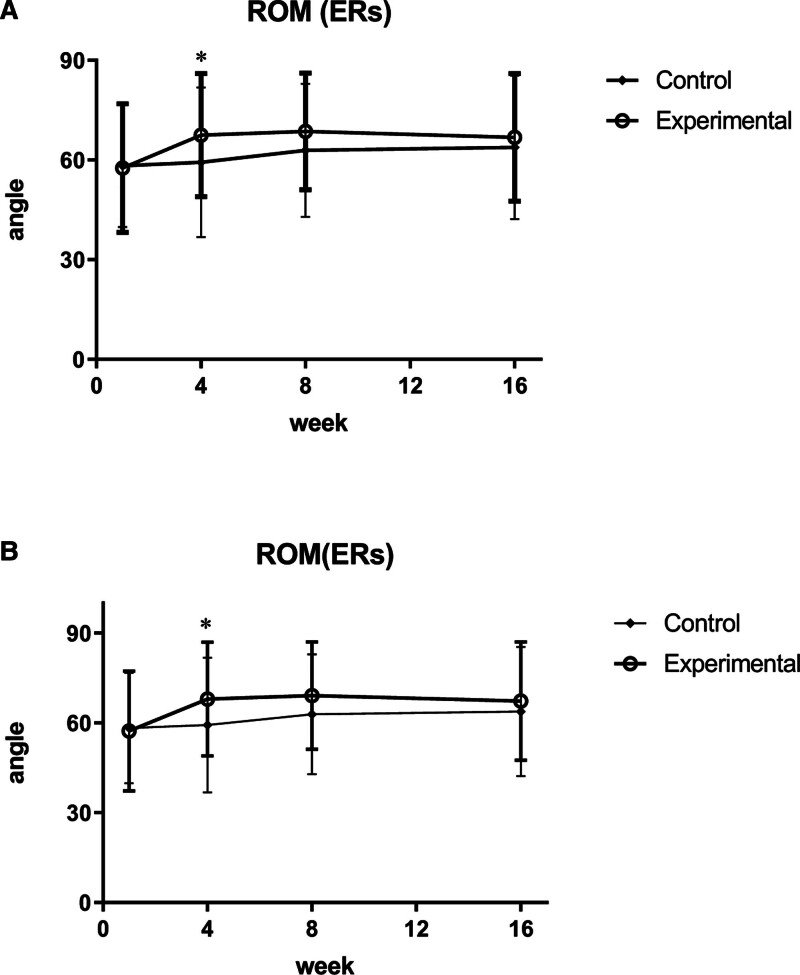
Changes in range of motion of external rotation side (ROM [ERs]). (A) ITT analysis group. (B) PP analysis group. *P < .05. ERs = external rotation side, ROM = range of motion.

The ROM of the ER side was significantly different between the groups at 4 weeks from baseline (visit 4; *P* = .046). The variance of the experimental group (9.88 ± 16.11) was considered to be clinically significant as compared to the control group (Fig. 6A). Other outcome measurements, such as VAS, SPADI, RC-QoL, and EQ-5D, showed significant results within the experimental group, which was expected to be an effect of TEA treatment, such as pain reduction, functional shoulder improvement, and quality of life at 16 weeks after baseline (visit 10). However, statistical significance was not observed between the groups. In the RC-QoL, sections C (recreational activities, sports participation, or competition concerns) and D (lifestyle concerns) showed statistical significance at visit 4; however, there was no statistical significance in the total RC-QoL at visit 4.

#### 3.3.2. PP analysis group

The ROM of the ER side was significantly different between the groups at 4 weeks from baseline (visit 4; *P* = .035). The variance of the experimental group (10.63 ± 16.37) was considered to be clinically significant as compared to the control group (Fig. 6B). Other outcome measurements, such as VAS, SPADI, RC-QoL, and EQ-5D, showed significant results within the experimental group, which was expected to be an effect of TEA treatment, such as pain reduction, functional shoulder improvement, and quality of life at 16 weeks after baseline (visit 10); however, statistical significance was not observed between the groups. In the RC-QoL, sections C (recreational activities, sports participation, or competition concerns) and D (lifestyle concerns) showed statistical significance at visit 4; however, there was no statistical significance in the total RC-QoL at visit 4.

### 3.4. Safety outcome

For safety evaluation, vital signs, laboratory test results, and AEs were evaluated in participants who received treatment at least once. Some AEs were reported during the trial, but no correlation with the treatment was found. Therefore, there was no evidence suggesting that it was clinically harmful.

### 3.5. Evaluation of cost-effectiveness

#### 3.5.1. Use ROM as an evaluation index

##### 3.5.1.1. ROM FF (full flexion)

ITT analysis showed that the ICER of the experimental group versus the control group was 4,16,404 won/ROM full flexion (USD 373.22/ROM FF). The TEA treatment was 202,300 won (USD 181.32) more expensive than the control treatment. However, considering the treatment of 8 weeks, an increase of 0.5° in ROM FF was observed in the experimental group. Since there was no socially agreed willingness to pay (WTP) for a 1° increase in ROM, it was impossible to determine which treatment was more economical. If the WTP for a 1° increase in ROM was more than 4,16,404 won (USD 373.22), TEA treatment could have been a better option (Table 4).

**Table 4:** Cost-effectiveness analysis results with ROM as an index (intention-to-treat).

ROM	Treatment option	Cost (won/dollar)	Incremental cost (won/dollar)	Effect (ROM, °)	Incremental effect (△ROM, °)	ICER (won/dollar)
FF	Control	₩4,70,773/$421.95	–	19.0	–	–
	Experimental	₩6,70,073/$600.59	₩2,02,300/$181.32	19.5	0.5	₩4,16,404/$373.22
ERs	Control	₩4,70,773/$421.95	–	4.5	–	–
	Experimental	₩6,70,073/$600.59	₩2,02,300/$181.32	11.3	6.8	₩29,586/$26.52
ER at 90	Control	₩4,70,773/$421.95	–	2.2	–	–
	Experimental	₩6,70,073/$600.59	₩2,02,300/$181.32	9.7	7.5	₩26,873/$24.09
IR at 90	Control	₩4,70,773/$421.95	–	14.9	–	Dominant
	Experimental	₩6,70,073/$600.59	₩2,02,300/$181.32	9.4	-5.6	Dominated
IRp	Control	₩4,70,773/$421.95	–	1.667	–	–
	Experimental	₩6,70,073/$600.59	₩2,02,300/$181.32	2.032	0.366	₩5,53,349/$497.76

The won/dollar exchange rate was 1115.7 won per dollar.

Control group: simple acupuncture.

Experimental group: thread-embedding acupuncture treatment.

ER at 90 = external rotation at 90°, ERs = external rotation side, FF = full flexion, ICER = incremental cost-effectiveness ratio, IR at 90 = internal rotation at 90°, IRp = internal rotation posterior, ROM = range of motion.

The PP analysis showed that the ICER of the experimental group versus the control treatment was 1,36,051 won/ROM FF (USD 121.94/ROM FF). The TEA treatment was 2,02,300 won (USD 181.32) more expensive than the control treatment. However, considering the treatment of 8 weeks, a 1.5° increase in ROM FF was observed in the experimental group. As there was no socially agreed WTP for a 1° increase in ROM, it was impossible to determine which treatment was more economical. If the WTP for a 1° increase in ROM was more than 1,36,051 won (USD 121.94 dollar), TEA treatment could have been a better option (Table 5).

**Table 5:** Cost-effectiveness analysis results with ROM as an index (per-protocol).

ROM	Treatment option	Cost (won/dollar)	Incremental cost (won/dollar)	Effect (ROM, °)	Incremental effect (△ROM, °)	ICER (won/dollar)
FF	Control	₩4,70,773/$421.95	–	19.0	–	–
	Experimental	₩6,70,073/$600.59	₩2,02,300/$181.32	20.5	1.5	₩1,36,051/$121.94
ERs	Control	₩4,70,773/$421.95	–	4.5	–	–
	Experimental	₩6,70,073/$600.59	₩2,02,300/$181.32	12.2	7.7	₩26,198/$23.48
ER at 90	Control	₩4,70,773/$421.95	–	2.2	–	–
	Experimental	₩6,70,073/$600.59	₩2,02,300/$181.32	10.2	8.0	₩25,208/$22.60
IR at 90	Control	₩4,70,773/$421.95	–	14.9	–	Dominant
	Experimental	₩6,70,073/$600.59	₩2,02,300/$181.32	10.6	-4.3	Dominated
IRp	Control	₩4,70,773/$421.95	–	1.667	–	–
	Experimental	₩6,70,073/$600.59	₩2,02,300/$181.32	2.034	0.368	₩5,50,002/$492.97

The won/dollar exchange rate was 1115.7 won per dollar.

Control group: simple acupuncture.

Experimental group: thread-embedding acupuncture treatment.

ERs = external rotation side, ICER = incremental cost-effectiveness ratio, IRp = internal rotation posterior, RC-QoL = rotator cuff quality of life score.

##### 3.5.1.2. ROM ERs (external rotation side)

ITT analysis showed that the ICER of the experimental group versus the control group was 29,586 won/ROM external rotation side (USD 26.52/ROM ERs). The TEA treatment was 2,02,300 won (USD 181.32) more expensive than the control treatment. However, considering the treatment of 8 weeks, a 6.8° increase in ROM ERs was observed in the experimental group. As there was no socially agreed WTP for a 1° increase in ROM, it was impossible to determine which treatment was more economical. If the WTP for a 1° increase in ROM was more than 29,586 won (USD 26.52), TEA treatment could have been a better option (Table 4).

The PP analysis showed that the ICER of the experimental group versus the control group was 26,198 won/ROM ERs (USD 23.48 dollar). The TEA treatment was 2,02,300 won (USD 181.32) more expensive than the control treatment. However, considering the treatment of 8 weeks, a 7.7° increase in ROM ERs was observed in the experimental group. As there was no socially agreed WTP for a 1° increase in ROM, it was impossible to determine which treatment was more economical. If the WTP for a 1° increase in ROM was more than 26,198 won (USD 23.48), TEA treatment could have been a better option (Table 5).

##### 3.5.1.3. ROM ER at 90 (external rotation at 90°)

ITT analysis showed that the ICER of the experimental group versus the control treatment was 26,873 won/ROM ER at 90 years (USD 24.09/ROM ER at 90 years). The TEA treatment was 2,02,300 won (USD 181.32) more expensive than the control treatment. However, considering the treatment of 8 weeks, a 7.5° increase in ROM ER at 90 was observed in the experimental group. As there was no socially agreed WTP for a 1° increase in ROM, it was impossible to determine which treatment was more economical. If the WTP for a 1° increase in ROM was more than 26,873 won (USD 24.09), TEA treatment could have been a better option (Table 4).

PP analysis showed that the ICER of the experimental group versus the control treatment was 25,208 won/ROM ER at 90 years (USD 22.60/ROM ER at 90 years). The TEA treatment was 2,02,300 won (USD 181.32) more expensive than the control treatment. However, considering the treatment of 8 weeks, an 8.0° increase in ROM ER at 90 was observed in the experimental group. As there was no socially agreed WTP for a 1° increase in ROM, it was impossible to determine which treatment was more economical. If the WTP for a 1° increase in ROM was more than 25,208 won (USD 22.60), TEA treatment could have been a better option (Table 5).

##### 3.5.1.4. ROM IR at 90 (internal rotation at 90°)

In the ITT analysis group, TEA treatment was less cost-effective than in the control group (dominated). Not only was TEA treatment 2,02,300 won (USD 181.32) more expensive in the experimental group, but the results were 5.6° higher in the control group after 8 weeks of treatment (Table 4).

In the PP analysis group, TEA treatment was less cost-effective than in the control group (dominated). Not only was TEA treatment 2,02,300 won (USD 181.32) more expensive in the experimental group, but the results were 4.3° higher in the control group after 8 weeks of treatment (Table 5).

##### 3.5.1.5. ROM IRp (internal rotation posterior)

ITT analysis showed that the ICER of the experimental group versus the control group was 5,55,349 won/ROM IRp (USD 497.76/ROM IRp). The TEA treatment was 2,02,300 won (USD 181.32) more expensive than the control treatment. However, considering the treatment of 8 weeks, a 0.366° increase in ROM IRp was observed in the experimental group. As there was no socially agreed WTP for a 1° increase in ROM, it was impossible to determine which treatment was more economical. If the WTP for a 1° increase in the ROM was more than 5,53,349 won (USD 497.76), TEA treatment could have been a better option (Table 4).

PP analysis showed that the ICER of the experimental group versus the control group was 5,50,002 won/ROM IRp (USD 492.97/ROM IRp). The TEA treatment was 2,02,300 won (USD 181.32) more expensive than the control treatment. However, considering the treatment of 8 weeks, a 0.368° increase was observed in the experimental group. As there was no socially agreed WTP for a 1° increase in ROM, it was impossible to determine which treatment was more economical. If the WTP for a 1° increase in ROM was more than 5,50,002 won (USD 492.97), TEA treatment could have been a better option (Table 5).

#### 3.5.2. Use RC-QoL as an evaluation index

ITT analysis showed that the ICER of the experimental group versus the control group was 4,27,880 won/RC-QoL (USD 383.51/RC-QoL). The TEA treatment was 2,02,300 won (USD 181.32) more expensive than the control treatment. However, considering the treatment of 8 weeks, a 0.5-point increase in RC-QOL was observed in the experimental group. Since there was no socially agreed WTP for a 1 point increase in RC-QOL, it was impossible to determine which treatment was more economical. If the WTP to increase 1 point in RC-QoL was more than 4,27,880 won (USD 383.51), TEA treatment could have been a better option (Table 6).

**Table 6:** Cost-effectiveness analysis results with RC-QoL as an index (intention-to-treat).

Treatment option	Cost (won/dollar)	Incremental cost (won/dollar)	Effect (RC-QoL)	Incremental effect (△RC-QoL)	ICER (won/dollar)
RC-QoL					
Control	₩4,70,773/$421.95	–	15.0	–	–
Experimental	₩6,70,073/$600.59	₩2,02,300/$181.32	15.5	0.5	₩4,27,880/$383.51

The won/dollar exchange rate was 1115.7 won per dollar.

Control group: simple acupuncture.

Experimental group: thread-embedding acupuncture treatment.

ICER = incremental cost-effectiveness ratio, RC-QoL = rotator cuff quality of life score.

PP analysis showed that the ICER of the experimental group versus the control group was 2,43,297 won/RC-QoL (USD 218.07/RC-QoL). The TEA treatment was 2,02,300 won (USD 181.32) more expensive than the control treatment. However, considering the treatment of 8 weeks, a 0.9-point increase in RC-QoL was observed in the experimental group. Since there was no socially agreed WTP for a 1 point increase in RC-QoL, it was impossible to determine which treatment was more economical. If the WTP to increase 1 point in RC-QoL was more than 2,43,297 won (USD 218.07), TEA treatment could have been a better option (Table 7).

**Table 7:** Cost-effectiveness analysis results with RC-QoL as an index (per-protocol).

Treatment option	Cost (won/dollar)	Incremental cost (won/dollar)	Effect (RC-QoL)	Incremental effect (△RC-QoL)	ICER (won/dollar)
RC-QoL					
Control	₩4,70,773/$421.95	–	15.0	–	–
Experimental	₩6,70,073/$600.59	₩2,02,300/$181.32	15.9	0.9	₩2,43,297/$218.07

The won/dollar exchange rate was 1115.7 won per dollar.

Control group: simple acupuncture.

Experimental group: thread-embedding acupuncture treatment.

ICER = incremental cost-effectiveness ratio, RC-QoL = rotator cuff quality of life score.

#### 3.5.3. Use EQ-5D-5L as an evaluation index

In the ITT analysis group, TEA treatment was less cost-effective than in the control group (dominated). Not only was TEA treatment 2,02,300 won (USD 181.32) more expensive in the experimental group, but the results were also 0.015 higher in the control group after 8 weeks of treatment (Table 8).

**Table 8:** Cost-effectiveness analysis results with EQ-5D-5L as an index (intention-to-treat).

Treatment option	Cost (won/dollar)	Incremental cost (won/dollar)	Effect (EQ-5D-5L)	Incremental effect (△EQ-5D-5L)	ICER (won/dollar)
EQ-5D-5L					
Control	₩4,70,773/$421.95	–	0.073	–	Dominant
Experimental	₩6,70,073/$600.59	₩2,02,300/$181.32	0.058	−0.015	Dominated

The won/dollar exchange rate was 1115.7 won per dollar.

Control group: simple acupuncture.

Experimental group: thread-embedding acupuncture treatment.

EQ-5D-5L = EuroQol 5-dimension 5 levels, ICER = incremental cost-effectiveness ratio.

In the PP analysis group, TEA treatment was less cost-effective than in the control group (dominated). Not only was TEA treatment 2,02,300 won (USD 181.32) more expensive in the experimental group, but the results were also 0.013 higher in the control group after 8 weeks of treatment (Table 9).

**Table 9:** Cost-effectiveness analysis results with EQ-5D-5L as an index (per-protocol).

Treatment option	Cost (won/dollar)	Incremental cost (won/dollar)	Effect (EQ-5D-5L)	Incremental effect (△EQ-5D-5L)	ICER (won/dollar)
EQ-5D-5L					
Control	₩4,70,773/$421.95	–	0.073	–	Dominant
Experimental	₩6,70,073/$600.59	₩2,02,300/$181.32	0.060	−0.013	Dominated

The won/dollar exchange rate was 1115.7 won per dollar.

Control group: simple acupuncture.

Experimental group: thread-embedding acupuncture treatment.

EQ-5D-5L = EuroQol 5-dimension 5 levels, ICER = incremental cost-effectiveness ratio.

## 4. Discussion

Based on the analgesic and continuous stimulatory effects of TEA on soft tissues, this clinical trial evaluated the effectiveness, safety, and cost-effectiveness of TEA in patients with chronic RCD. We established detailed diagnostic criteria in previous studies.^[31]^ A randomized controlled clinical trial was then conducted with participants meeting these criteria.

In the effectiveness assessment of the primary outcome (VAS), there was no statistically significant difference; however, a decrease in the pain score was observed within the groups. These differences were more pronounced in the TEA group than in the STEA group. Despite the statistical significance, TEA treatment was considered to have an effect on pain reduction. In the assessment of secondary outcomes, only the ROM of the ER side showed statistical significance as compared to the STEA group (*P* < .05). Other factors also showed improvement, but were not statistically significant. In the safety evaluation, no AEs were related to intervention. TEA is well-known for its medicinal properties in Korea; its safety has been empirically proven. Therefore, it was anticipated that no hazards were found in this study and that TEA can be considered safe. Based on these results, it can be applied to chronic RCD in clinical practice without any hazards.

In the cost-effectiveness evaluation, it was found that TEA treatment might be more beneficial than the control treatment, according to some indices as well as the socially agreed WTP based on those indices. When ROM IR at 90° and EQ-5D-5L were used as indices, the experimental group did not exhibit a better effect than the control group; the average cost was also higher in the control group. However, in other indices, such as ROM (FF, ERs, ER at 90, and IRp) and RC-QoL, TEA was more economical than the control group; therefore, depending on the WTP for these indices, TEA treatment might be more economical than the control treatment. If the WTP for each index may be obtained in the future, the cost-effectiveness of TEA treatment for chronic RCD can be determined; however, additional research is needed.

Although no significant results were found, other than an improvement in the ROM of the ER side, this study had several advantages. First, this was a randomized controlled trial. The STEA treatment was used as a control to blind the participants and to prevent needle-specific physiological effects. The participants could not receive treatment with the practitioner blinded; therefore, patient-assessor blinding was applied. Considering the physiological effects of needle insertion, sham needles of the same form were used to obtain identical physiological effects. Second, the diagnostic criteria for participant selection were established through expert discussions and previous research. The criteria used in past studies on chronic RCD to evaluate the effect of acupuncture treatment remain unclear. Third, the effects of the TEA were assessed comprehensively using various indicators. Although the main purpose of shoulder disease treatments was to reduce pain, improving shoulder function and quality of life remain crucial. Therefore, we used various indicators to evaluate pain, shoulder function, and quality of life, in order to assess the effects of TEA on various aspects of daily life.

Despite these advantages, there are also limitations to this study. First, considering the characteristics of the polydioxanone thread in TEA, which remained approximately 6 months after insertion into the body and has a 50% of tensile force after 4 weeks, the results should be tracked from 4 weeks to 6 months.^[44,45]^ However, no observations were made after 16 weeks of the intervention; therefore, the investigator could not discuss the long-term effects of TEA. To assess the mid- to long-term effectiveness of TEA, studies should observe long-term follow-up processes of at least 4.^[46]^ Second, although statistical significance was not observed between the groups, except for the ROM of the ER side, the effectiveness of acupuncture itself cannot be ignored, as other outcome measurements showed improvement within each group. Acupuncture alone has been reported to be effective in improving pain. The needle-specific physiological effects of STEA itself could have contributed to pain improvement to some extent. Third, while various assessment tools were used to evaluate TEA efficacy, their limitations warrant consideration. The SPADI may exhibit ceiling effects in high-functioning patients, noninterchangeable subscale scores, limited sensitivity to occupational or recreational disability, inconsistent cross-cultural validation, and potential measurement error with repeated use. Similarly, the EQ-5D is subject to ceiling and floor effects, lacks country-specific value sets in some regions, provides limited clinical detail, and may inadequately capture the impact of certain conditions or treatments on patient well-being. Fourth, although common differential diagnoses such as acromioclavicular osteoarthritis, glenohumeral instability or osteoarthritis, superior labrum anterior-to-posterior tear, suprascapular nerve injury, Parsonage-Turner syndrome, syringomyelia-related neuropathic shoulder, and fibromyalgia were considered, their complete exclusion could not be ensured. Lastly, recruitment through public advertisements further raises concerns about diagnostic accuracy, even with screening by a Korean Medicine Doctor specializing in musculoskeletal disorders and acupuncture, and the potential for misclassification of chronic RCD cannot be entirely ruled out. Also, practitioners were not blinded, and despite restrained communication with participants, some bias was inevitable.

Nevertheless, this clinical trial is meaningful because no previous randomized controlled trials have used TEA for chronic RCD. The results of this study will provide physicians with information regarding the clinical use of TEA for the treatment of shoulder pain. We have provided important information about the sample size, inclusion and exclusion criteria, outcome measurements, and economic evaluation data, which may be applicable to further studies. Moreover, the safety of TEA enables TEA treatment to be a useful method for treating diverse diseases. Therefore, this study provides important data for designing further studies on shoulder diseases and TEA treatment. This study, based on an actual clinical environment, can serve as a good precedent for future research and development of Korean medicine treatments for the management of patients with RCD.

## 5. Conclusion

After applying TEA to chronic RCD, no statistically significant differences were found, except for ROM (ERs). However, pain reduction, improvement in function, and quality of life in the TEA group were numerically better than those in the STEA group; therefore, TEA could be applied to patients with shoulder pain and decreased quality of life. In the safety assessment, there were no safety hazards associated with TEA. In addition, according to some indices, TEA was more economical than simple acupuncture. Further research, with an extended trial period or increased sample size to verify the long-term effects of TEA, is expected to provide further information to demonstrate the therapeutic and economic effects of TEA treatment.

## Acknowledgments

We sincerely thank Yong-Hyeon Baek, the corresponding author. We would also like to thank all the researchers and participants who agreed to participate in this study.

## Author contributions

**Conceptualization:** Yeon-Cheol Park.

**Data curation:** Man S. Kim.

**Formal analysis:** Yeon-Cheol Park, Sangyeup Chae, Yeonhak Kim, Yoonsung Lee.

**Funding acquisition:** Yong-Hyeon Baek.

**Investigation:** Yeon-Cheol Park.

**Methodology:** Yeon-Cheol Park, Byung-Kwan Seo.

**Project administration:** Yeon-Cheol Park.

**Resources:** Byung-Kwan Seo.

**Software:** Yoonsung Lee, Man S. Kim.

**Supervision:** Yong-Hyeon Baek.

**Validation:** Yeon-Cheol Park, Yong-Hyeon Baek.

**Visualization:** Sangyeup Chae, Yeonhak Kim, Byung-Kwan Seo.

**Writing—original draft:** Sangyeup Chae.

**Writing—review & editing:** Yeon-Cheol Park, Sangyeup Chae, Yeonhak Kim, Yoonsung Lee, Man S. Kim.
